# Fluoride Varnishes for Preventing Occlusal Dental Caries: A Review

**DOI:** 10.3390/dj9060064

**Published:** 2021-06-03

**Authors:** Alaa Baik, Najlaa Alamoudi, Azza El-Housseiny, Amani Altuwirqi

**Affiliations:** 1 Pediatric Dentistry Department, King Abdulaziz University Dental Hospital, Jeddah 21589, Saudi Arabia; ambaik@kau.edu.sa; 2 Pediatric Dentistry Department, Faculty of Dentistry, King Abdulaziz University, Jeddah 21589, Saudi Arabia; aaltuwirqi@kau.edu.sa; 3 Pediatric Dentistry Department, Faculty of Dentistry, Alexandria University, Alexandria 21526, Egypt; aalhosseiny@kau.edu.sa

**Keywords:** fluoride varnish, occlusal caries, deep fissures, caries prevention, topical fluoride, cariostatic agents

## Abstract

Dental caries are most likely to occur on occlusal surfaces from the early eruptive stages of the tooth. In children, about 80% to 90% of dental caries are occlusal caries. Different preventive modalities are used to prevent occlusal caries. One of the methods used for occlusal caries prevention is fluoride varnish. A vast number of clinical trials have evaluated several types of sealant material and fluoride varnish to assess their ability to prevent occlusal caries in both primary and permanent dentition. The purpose of the current study was to provide an updated overview of the development, composition, mechanism of action, application, and safety of fluoride varnish, as well as its effect on occlusal caries prevention. This review of recently published studies on fluoride varnish and its effect on occlusal caries prevention shows that in children at moderate to high risk of caries, fluoride varnishes prevent occlusal caries. Both resin-based fissure sealants and fluoride varnish are effective for occlusal caries prevention for first-permanent molars. However, it was not possible to identify which one has the best effect. It is recommend that fluoride varnish is applied for children at high risk of caries two to four times per year.

## 1. Introduction

Tooth decay is one of the most common microbiological infectious diseases worldwide. The World Health Organization (WHO) estimated that 60% to 90% of all children of school age have experienced dental caries [1]. Dental caries is a multifactorial disease affecting both children and adults that leads to demineralization of the tooth structure. The main causative factors include the presence of endogenous cariogenic bacteria, frequent consumption of fermentable carbohydrates, and a susceptive tooth and host [2].

Although dental caries can progress if left untreated, it can be prevented, reversed, or arrested at its initial stages [3]. Prevention and minimal intervention treatment following early detection of the lesion are becoming the new philosophy for managing dental caries. The antibacterial agent chlorhexidine has shown a preventive effect against dental caries. Additionally, diet and fluoride have been shown to contribute to the arrest and prevention of dental caries [4].

In children and young adults, dental caries is most likely to occur at occlusal surfaces from the early eruptive stages of the tooth [5,6,7,8,9]. About 80% to 90% of dental caries cases in children are occlusal caries [7,10]. First molars are the most susceptible teeth to caries, followed by second molars [11,12].

The enamel of the tooth is exposed to various demineralization–remineralization cycles throughout the day. The balance between remineralization processes and demineralization processes regulates whether dental caries remain static, progress, or are reversed [13].

Biomineralization is a complex dynamic process that occurs throughout life. Demineralization of teeth is caused by acids from foods and drinks and microbial attack of bacteria [14]. These acids lead to plaque pH reduction and subsequent chemical dissolution of both the organic and inorganic matrix and mineral loss of the enamel. The phosphate ions and calcium ions disperse into the oral cavity, resulting in enamel demineralization [15].

However, this can be reversed and arrested if the lesion is detected before cavitation and if the number of cariogenic bacteria is reduced. During demineralization, calcium ions and phosphate ions move from the tooth to the saliva and the opposite occurs during remineralization. Phosphate and calcium ions diffuse into the enamel surface and form fluorapatite. Fluorapatite can resist demineralization and further enhance remineralization [15]. Furthermore, remineralization agents such as synthetic hydroxyapatite can inhibit demineralization and enhance remineralization. Hydroxyapatite can occlude dentin tubules and attach to tooth surfaces, thus enhancing remineralization [16].

Several factors can affect this cycle, including patient education, reducing the rate of free sugar intake, controlling plaque formation, improving the buffering capacity of saliva, enhancing the salivary flow rate, and using additional caries preventive measures such as antibacterial agents and fluoride [17].

Many fluoride systems that supply the tooth surfaces with fluoride are used in dentistry. Fluoride is available in two forms: a systemic form and a topical form [18]. Topical fluoride systems are available for use at home with a low concentration of fluoride or professionally with a high concentration of fluoride.

Systemic fluoride is available via water fluoridation or can be taken in through the diet or fluoride supplements. Ever since the establishment of water fluoridation seven decades ago, fluoride has been at the center of caries preventive approaches [19]. The systemic form of fluorides is the form that can be ingested. Systemically ingested fluoride integrates with the tooth elements and structure during the formation of the teeth before their eruption. It can change hydroxyapatite into fluorapatite, thus making the teeth more resistant to caries [20].

Topical home fluoride is available as mouth rinses, gels, and dentifrices (tubes of toothpaste). High-concentration professionally applied fluoride is available in the dental office as gels, foam, and varnish. Frequent exposure to a low concentration of topical fluoride is associated with caries reduction [21].

Topical fluorides can be used to enhance remineralization. Topical fluorides increase the availability of fluoride ions in saliva and enhance the formation of fluorapatite. Fluorapatite is strongly resistant to acid attacks and demineralization. Additionally, increasing the concentration of fluoride may inhibit bacterial metabolism [22].

Varnish is a professional topically applied fluoride system. Varnish has been used extensively as a caries prevention strategy for over 3 decades. Topical varnishes slowdown the release of active substances such as chlorhexidine, oxidative agents from bleaching systems, and fluoride [23].

Fluoride varnishes (FVs) are professionally applied fluoride treatments that are usually applied two or four times per year depending on an individualized caries risk assessment of a child. This varnish can stick to the tooth surfaces for a relatively long time, thus releasing fluoride in an efficient and effective way [23].

In the 1960s, dental sealants were developed to reduce and prevent pits and fissures of occlusal caries. The use of sealants was increased afterwards. Sealant works as a physical barrier to seal deep fissures and prevent food and bacterial accumulation and subsequent caries formation. Ideal sealants require proper isolation and etching prior to application of the sealant materials. Sealant application is effective for reducing caries. When the caries rate of sealed teeth and controlled unsealed teeth was compared, an 87% reduction in caries after one year was found in the sealed group. Moreover, a 60% reduction in caries at 4–4.5 years after treatment with sealant was found [24]. However, the success rate of the sealant was technique-sensitive and highly dependent on the clinical application and the cooperation of the patient. The etching and drying step is critical and can adversely affect the longevity of the sealant if it is not done properly. Many steps are required to deliver an ideal sealant treatment. In contrast, FV application is simple with fewer steps and less sensitivity to moisture. Several studies have investigated the effectiveness of fluoride varnish in inhibiting occlusal caries and compared it with fissure sealant. Moreover, studies have investigated whether FV is a suitable alternative to sealant (in terms of patient acceptability, cost clinical criteria) [25].

The purpose of this review is to give an updated overview of the development, composition, mechanism of action, application, and safety of fluoride varnish and to assess its role in preventing dental occlusal caries compared to no treatment or treatment with fissure sealant.

## 2. Materials and Methods

A web search for English papers published from 1990 to 2020 was conducted. Different search engines were used, including Google Scholar, PubMed, online review, and the Cochrane library database. A total of 55 studies were included in this review. Articles that included a description of the fluoride varnish composition and the advantages, disadvantages, and mechanism of action of fluoride varnish were included in the study. Additionally, studies that described the effect of fluoride varnish in caries prevention in children were included. A total of 23 studies were excluded. Studies that described the effect of fluoride varnish on tooth sensitivity, enamel erosion, treatment of proximal incipient caries, and white spot lesions were not included in this review. The following keywords were used: fluoride varnish, occlusal caries, caries prevention, fissure sealant, fluoride, and topical fluoride.

## 3. Results and Discussion

One hundred and sixty studies were retrieved. Their references were also reviewed to obtain more related studies. Duplicated copies were excluded. In the current literature review, 61 studies were included.

### 3.1. Overview of Fluoride Varnishes

In 1964, professionally applied fluoride varnishes were developed by a German researcher called Schmidt, who used them to prevent dental caries. The varnishes were developed to maximize the time of fluoride exposure to overcome the disadvantages of existing topical fluoride systems, such as fluoridated mouth rinses and gels, by increasing the adherence of fluoride to the enamel of the teeth and thus extending the duration of uptake [23].

Nowadays, more than thirty fluoride-containing varnishes are available on the market. There are a vast variety of delivery systems and compositions that can show variable pharmacokinetics [26]. Even though there are diverse fluoride varnishes, there are two main forms. The first of these, which was developed in 1960, is sodium fluoride (NaF) (Duraphat, Colgate Oral Pharmaceuticals, New York, NY, USA), and the second is fluoride Silane (Fluor Protector, Ivoclar Vivadent, Schaan, Liechtenstein), which was developed in 1975 [27].

In 1994, fluoride varnish was approved by the Food and Drug Administration (FDA) for use as a root desensitizer and cavity liner. The first fluoride varnish approved by the FDA was Duraphat (Colgate Oral Pharmaceuticals, New York, NY, USA), and since then, its use and development have been increasing dramatically [23]. Nevertheless, the FDA did not approve fluoride varnish for use as an anti-caries agent. Thus, its use as an agent to reduce caries is considered off-label. Despite this, the FDA regulation did not prevent clinicians from using legally available drugs (varnish) as caries preventive measures when they are the best option for the patient, based on clinical professional judgment and knowledge [23].

The application and spreading of varnishes on tooth surfaces are usually done with cotton pellets, small brushes, or syringes with or without prophylaxis of the teeth. It is recommended that varnish is applied two to four times per year. Despite their high concentration of fluoride, fluoride varnishes are considered safe. This is may be due to the fast setting of the varnish, which reduces the amount of fluoride ingested. Additionally, the prolonged contact time between fluoride varnish and enamel leads to slow release of the fluoride [19].

However, conventional fluoride varnish requires multiple applications to provide an anti-caries effect [28]. Accordingly, light-curable fluoride varnish (LCFV) was developed as a localized protective varnish for both dentin and enamel tooth surfaces. It has shown superior advantages in terms of sustainability and longevity compared with conventional fluoride varnish [29].

Light-curable resin-modified glass ionomer (RMGI) varnishes, such as Vanish XT varnish and Clinpro XT varnish “(Vanish™ XT Extended Contact Varnish, 3M ESPE, St. Paul, MN, USA)” were introduced into the market in 2009. These varnishes have a slow fluoride releasing property, allowing their effect to last for up to six months [30].

Vanish XT varnish is used to treat hypersensitive teeth and as a sealing material for surfaces of teeth that have an increased caries risk, such as recently or partially erupted teeth, tooth surfaces adjacent to orthodontic brackets, erosion from acid, and non-cavitated lesions [30]. The glass ionomer composition of Vanish XT varnish enhances the tooth structure adhesion and retains fluoride. Additionally, Vanish XT varnish can release calcium and phosphate [31]. Several studies have found that light-curable RMGI varnishes result in enamel demineralization prevention for longer than fluoride varnish [29].

### 3.2. Composition

The original composition of fluoride varnish has changed over time, and the current fluoride varnishes used in the United States having the following active ingredients: 5% NaF with a rosin and ethanol carrier in both Duraphat (Colgate-Palmolive, New York, NY, USA) and Duraflor (Medicom, Montreal, QC, Canada) [32]. Fluor protector (Ivoclar Vivadent, Schaan, Liechtenstein) contains 0.9% difluorosilane on a polyurethane varnish base with ethyl acetate and isoamyl propionate solvents [32].

Duraphat contains 5% sodium fluoride (NaF) by weight or 22,600 parts per million fluoride ions (ppm F). It contains 2.26% fluoride in the natural carrier resin with alcohol listed as a solvent. Fluor protector contains 0.9% difluorosilan by weight or 1000 ppm F in polyurethane-based varnish. When it was originally developed in 1975, its fluoride concentration was 0.7%. However, the concentration was changed to 0.1% in 1987 [32]. Duraphat comes in a 10 mL tube that is suitable for multiple uses, while fluor protector comes in an ampule that is used for a single (0.4 mL) or multiple doses (1.0 mL) (Table 1).

The main components of most varnishes are similar with just a few differences. They are composed of resin (colophony), alcohol, and sodium fluoride. Alcohols, such as ethanol or others, are utilized as solvents to keep the varnish in a fluid form to enhance easy application. When the varnish comes into contact with air, the solvents evaporate and the varnish becomes adherent to the tooth surfaces, thereby increasing the duration of fluoride exposure [33].

In addition to these three main ingredients, other compounds may be present, such as stabilizing agents, adhesion-promoting agents, colorants, modifying agents, and flavoring agents [33]. Recently, to enhance remineralization, calcium–phosphate compounds were added to some fluoride varnishes. Amorphous calcium phosphate (ACP) was added to Enamel Pro (Premier Dental), while tri-calcium phosphate (TCP) was added to Vanish (3M ESPE). 

### 3.3. Fluoride Varnish Application

Fluoride varnishes are capable of closely attaching to enamel for several hours. Prophylaxis is not required prior to application, and tooth brushing can be adequate for teeth cleaning [34]. During application, the clinician uses different kinds of applicators, such as a syringe applicator, cotton-tip applicator, or brush, to apply 0.3 to 0.75 milliliters of varnish to the tooth enamel surfaces. To ensure the varnish is applied to interproximal areas, dental floss can be used. The application of fluoride varnish can take one to four minutes. The time required is related to the number of teeth found in the oral cavity. Drying teeth prior to varnish spreading is not mandatory, and teeth wiping using cotton rolls or gauze is considered adequate. To achieve the best outcome by increasing the contact time of the varnish with the teeth, the following instructions should be given to patients: After varnish application, patients should be instructed to avoid eating for two to four hours. Moreover, patients should be instructed not to brush their teeth on the night after application. It has been proven that varnish stays on the enamel surface for several hours and could stay there for a few days after application. In an in vitro study that evaluated the enamel surface microscopically after varnish application followed by a demineralization challenge, small blocks of varnish remained attached to enamel surfaces [35].

### 3.4. Advantages of Fluoride Varnish

Fluoride varnish has many advantages when compared with other topical fluorides. Varnishes can set rapidly when applied to enamel surfaces. They can stay attached to the surface for a long time—up to days. The fluoride slow-releasing property allows teeth to be exposed to a higher concentration of fluoride. The varnish application does not require thorough drying of the teeth; thus, it is simple, fast, and not technique sensitive. Additionally, prior prophylaxis is not mandatory; therefore, the chairside application time is short. Due to the rapid setting time of varnishes, they can be used with young children, special needs children, and patients with gagging reflux [34,36].

### 3.5. Disadvantages and Safety of Fluoride Varnish

Sodium fluoride varnishes can temporarily change the color of teeth. This color change is temporary and lasts for 24 h until the outer layer of the varnish is removed through brushing. A layer of varnish remains attached to the tooth for protection. Patient varnish acceptability varies from person to other. Most patients accept the presence of varnish on their teeth. However, some patients dislike the taste or the feeling of the sticky varnish layer on their teeth. A reapplication of fluoride varnish is required to maintain its preventive effect [19].

Colophony is a contact sensitizer found in different household agents (such as nail varnish cosmetics, chewing gum, and sticking plasters) as well as some dental materials such as fluoride varnish. Although uncommon, individuals with a known sensitivity to colophony (rosin) can develop allergic reactions to fluoride varnish. A burning sensation in the gingival tissue can occur with some patients. An allergic reaction can also occur if the patient has a contact allergy to colophony. Additionally, the dentist can develop an allergy to the colophony present in varnish following long-term exposure. Stomatitis or dermatitis can occur if the mucosa or skin of a hypersensitive individual is exposed directly to colophony (from varnish) [37]. The use of fluoride varnish in patients with ulcerative gingivitis or stomatitis is contraindicated [38].

Many fluoride sources that supply the tooth surface are available. Fluoride is available in two forms: systemic and topical [18,39]. Systemic fluoride is available via water fluoridation or through ingestion in the diet or in fluoride supplements [39,40]. Topical fluoride is available as mouth rinses, gels, dentifrices, foam, and varnish. The main source of human exposure to fluoride is water. Foods contain low levels of fluoride. The total fluoride intake should not exceed the potential toxic dose for fluoride (5 mg/kg) [39].

The use of fluoride varnishes is considered safe. After application of topical fluoride, a residual quantity of fluoride is found in the oral cavity. This residual fluoride can be swallowed, leading to an elevation of plasma fluoride levels [41]. However, the amount of remaining fluoride can be reduced by proper application, suction, and isolation during application and by following the instruction to expectorate for one minute after application [42].

The high fluoride level in Duraphat fluoride varnish (as high as 2.3%) may raise safety concerns. However, varnishes have a fast setting time and the property of slow fluoride release. These properties could be the reason for the lower peak level of plasma fluoride when compared with the plasma fluoride level following the application of fluoride gels [43].

Similar findings were observed when the parotid saliva peak level was measured following the application of fluor protector (Ivoclar Vivadent, Schaan, Liechtenstein) and Duraphat (Colgate Oral Pharmaceuticals, New York, NY, USA). Due to the low fluoride concentration of fluor protectors, the plasma fluoride level was lower in patients who received fluor protectors than in patients who underwent Duraphat application [44].

In general, fluoride varnishes are considered safe and acceptable by patients [34,45]. Randomized clinical trials did not report any side effects, serious short-term effects, or safety concerns following fluoride varnish application for children or infants [46,47]. Due to the rapid setting time of varnishes, which leads to little fluoride ingestion, the risk of acute toxic reactions following varnish application is considered minimal [23,48].

### 3.6. Mechanism of Action

When fluoride was first introduced to the market for caries prevention, it was believed that the action of fluoride during tooth formation was systemic in the pre-eruptive tooth stage. It was believed that fluoride contributed to tooth development and would lead to the formation of a less soluble enamel structure. However, following the high number of studies conducted in this area to understand the caries preventive role of fluoride, this belief has changed. Nowadays, the mechanism by which fluoride reduces dental caries is thought to be mainly post-eruptive (topical) [49].

In an attempt to understand the mechanism of action by which fluoride varnishes aid in caries prevention, it is important to first explore the overall mechanism of action of topical fluoride on teeth. Topical fluoride plays important roles in bacterial inhibition, fluorapatite formation, demineralization reduction, and remineralization enhancement [50,51].

#### 3.6.1. Bacterial Inhibition

Fluoride acts on the acids of oral bacteria such as *Mutans Streptococci* (*MS*) and reduces the formation of these acids. This effect is considered a topical effect that leads to a reduction in tooth colonization by bacteria and thus leads to a decrease in enamel demineralization. In vitro studies have suggested that the inhibitory impact of fluoride ions on oral bacteria is related to bacterial enzyme inhibition [52]. However, despite the multiple reviews and studies that have suggested that there is an antimicrobial influence of fluoride on oral bacteria metabolic activity, the exact relationship between oral biofilms and fluoride remains ambiguous [53].

The influence of topical fluoride Duraphat varnish (Colgate Oral Pharmaceuticals, New York, NY, USA) on the level of *Streptococcus mutans* (*S. mutans*) in dental plaque and saliva was investigated in school children. Buccal plaque and salivary samples were collected prior to varnish application and at four, ten, and twenty-one days after application. No significant reduction in *S. mutans* was found. Therefore, it was suggested that an increase in the fluoride concentration may not reduce the concentration of *S. mutans*, but rather, it may inhibit the metabolic activity of bacteria [54]. In another study, the influence of fluor protector varnish (Ivoclar Vivadent, Schaan, Liechtenstein) on the concentration of *S. mutans* present in the plaque of children with no caries was investigated [55]. It was found that the varnish resulted in a significant reduction in the plaque *S. mutans* count (*p* = 0.000). The authors demonstrated that the reduction in the bacterial count in their study could be related to the high fluoride concentration in fluor protector (consists of 0.1% fluoride, which is 1000 ppm). This fluoride could lead to the inhibition of various cellular processes by entering bacterial cells. However, this could be related to the adherence properties of the varnish. The coating layer of the varnish prevented *S. mutans* from adhering to the enamel surface.

#### 3.6.2. Fluroapitate Formation

During exposure of the tooth to fermentable carbohydrates, plaque bacteria secrete organic acids, which leads to demineralization of the colonized tooth surface. These acids lead to the dissolution of calcium phosphate (hydroxyapatite) in the dentin or enamel [56]. The bacterial acids cause a decline in the pH of the saliva and increase the hydrogen ion concentration, leading to enamel dissolution. The dissolved phosphate and calcium leach out of the enamel surface to the oral cavity.

This mineral loss can be reversed by preventive factors such as salivary flow, the levels of phosphate, calcium, and proteins in saliva, and the fluoride concentration in plaque and saliva. The dynamic process of remineralization and demineralization is distinguished by the flow of phosphate ions and calcium ions out of and into the enamel [56].

The topical fluoride application of a low-concentration fluoride system (such as dentifrices and mouth rinses) is assumed to result in ion exchange between hydroxyapatite (Ca_10_ (PO_4_)_6_ (OH)_2_) and the fluoride system. The reaction between a low level of fluoride and hydroxyapatite leads to the replacement of hydroxyl ions in the crystal by fluoride. If the hydroxyl groups (OH– ions) in hydroxyapatite are completely substituted by fluoride ions (F–), the resulting mineral is fluorapatite (Ca_10_ (PO_4_)_6_ F_2_) [17]. These fluorapatite crystals are a more stable structure than hydroxyapatite. However, the complete substitution of hydroxyl groups, even in severe fluorosis of the enamel, is never achieved [57]. Only ten percent of the hydroxyl groups can be replaced by fluoride on the enamel surface. This partial replacement of the hydroxyl positions by fluoride ions will lead to the formation of the hydroxyapatite–fluorapatite compound. This flurohydroxyapatite is a richer crystal. It is more stable, more acid resistant, and has overall lower solubility [17].

In addition to the ion exchange of fluoride and hydroxyl groups following long-term exposure to low levels of fluoride, through systems such as dentifrices and mouth rinses, fluorapatite crystal growth can also be a mechanism of fluoride reaction when the saliva is supersaturated with fluoride [58].

The third mechanism of fluoride reaction with apatite is calcium fluoride formation, which is the only outcome that occurs on enamel during short fluoride exposure to agents with high fluoride concentration levels, such as NaF or acidulated phosphate fluoride (APF) [59,60]. However, fluoride can also be adsorbed onto enamel hydroxyapatite crystals [61]. Both calcium fluoride and adsorbed fluoride are alkali-soluble [59]. These alkali-soluble fluorides are also named loosely bound fluoride and are referred to as calcium fluoride. The calcium fluoride attached to the enamel surface acts as a reservoir to release fluoride to the saliva, and plaque is gradually released later when the tooth is exposed to acid attacks. This fluoride inhibits demineralization of the tooth structure and decreases the susceptibility of the tooth to caries by different proposed mechanisms. The ions dissolve into hydroxyapatite and subsequently fill or replace the hydroxyl ion gaps in the crystal, thus stabilizing and reducing the solubility of the crystal structure [34].

There are contradictory findings regarding the chemical structure of fluoride and the amount of retained fluoride on tooth surfaces after varnish application. One in vivo study found a high fluoride concentration in the enamel surface one week after the application of Duraphat and they relied on it for the retention of fluoride in the form of fluorapatite [62]. Additionally, in 1983, Retief et al. suggested that the major reaction product found after in vitro application of Duraphat to enamel surfaces was fluorapatite [63]. They proposed that the adhesiveness of varnish might lead to a slow release of fluoride that favors fluorapatite formation. In contrast, other recent findings suggest that calcium fluoride is the main precipitated structure when enamel is treated with Duraphat [64,65,66].

A study done by Ogaard et al. explored fluoride retention in sound enamel and demineralized enamel after the application of Duraphat fluoride varnish. They aimed to detect whether the cariogenic reduction effect of fluoride involves the formation of fluorapatite (alkali-insoluble) or calcium fluoride (CaF2), which is a more alkali-soluble structure [65]. The controlled sound enamel was compared with demineralized teeth in terms of the amounts of CaF2 and fluorapatite. In the sound enamel, a significant fluoride level was found in the two superficial layers, and the plurality of fluoride formation was CaF2 ≥ 52%, whereas that of fluorapatite was ≤16.6%. Moreover, the demineralized enamel was found to hold significantly more fluoride in all three layers with 75% of the fluoride being in the form of fluorapatite [65].

Additionally, Cruz et al. suggested that no firmly bound fluoride (fluorapatite) was found immediately after Duraphat fluoride application [67]. They suggested that calcium fluoride structures act as a source of fluoride for fluorapatite formation. This fluorapatite phase is formed during remineralization within many pH cycles in plaque, and it does not occur with topical application. The firmly bound fluoride found after Duraphat (and 2% NaF) application [62,65,68] is most likely formed subsequently [67].

#### 3.6.3. Inhibition of Demineralization/Enhancing Remineralization

Remineralization is a natural process that occurs in the oral cavity. Saliva plays a major role in the remineralization process. The process takes place when the saliva is supersaturated with ions, allowing mineral precipitation. The balance between mineral absorption and loss is determined by the saturation degree of the saliva [69].

The level of fluoride ions in normal saliva is not optimal. However, normal saliva is supersaturated with phosphate and calcium ions. Fluoride ions are essential for caries prevention. However, fluoride ions cannot inhibit demineralization and reprecipitation of the minerals into enamel crystals by themselves. The bioavailability of phosphate ions and calcium ions in plaque is also essential for maintaining the integrity of structural hydroxyapatite crystals [70].

It has been proven that fluoride can enhance the tooth remineralization rate and re-precipitation of phosphate and calcium ions onto the enamel surface [65]. Fluoride adherence to nondemineralized tooth enamel by itself can provide a shield against bacterial acid attacks and thus prevent demineralization and promote remineralization. This can occur by attracting calcium ions and phosphate ions and stimulating mineral growth. Long-term exposure to low fluoride sources, such as water fluoridation, can be a beneficial factor for this process [56].

However, the mechanism is different with concentrated fluoride systems such as varnishes. As previously stated, the application of agents containing higher fluoride concentrations leads to the formation of an intermediate product, calcium fluoride, on the tooth surface, on demineralized enamel, and in plaques [65,71]. These calcium fluoride structures are seen as small globules on the tooth surface following varnish application under a scanning electron microscope (SEM) [72]. During the process of calcium fluoride formation on tooth surfaces, phosphate ions are attached to these globular structures to stabilize them [73]. For a long time, calcium fluoride formation was considered an unfavorable side reaction of fluoride exposure due to its similar solubility in saliva as in water [74]. Nonetheless, many studies have proven that in neutral-pH saliva, calcium fluoride is quite insoluble and can stay attached to enamel surfaces for several weeks or months after the topical application of fluoride. They speculated that the resistance of calcium fluoride might be due to the attachment of phosphate ions to calcium sites present at the calcium fluoride crystal surface under a neutral pH [75,76,77].

Calcium fluoride acts as a reservoir of fluoride ions and a site of subsequent release of fluoride under the low pH conditions of plaque. The released fluoride stops the outward movement of phosphate and calcium from the enamel and diffuses with the acid from plaque into the enamel, efficiently enhancing the rate of remineralization and inhibiting demineralization. Phosphate ions protect the calcium fluoride once more when the pH of the plaque is reversed to normal, and this continues until the next decline in pH [17].

Areas with high susceptibility to caries such as pits and fissures, proximal surfaces, and incipient carious lesions have great surface areas. Fluoride varnishes tend to contain more calcium fluoride deposits at these large areas where fluoride is most needed [78]. Fluoride varnish sticks to enamel surfaces for a long time when compared with other forms of professional fluoride such as gels. The varnish keeps in close contact with the teeth for several hours or even days after application [65]. The effects of varnish are considered superior to those of other topical concentrated fluorides in pits and fissures because of its prolonged adhesiveness and greater uptake of fluoride in both permanent and primary dentition [79]. The greatest release of fluoride ions occurs in the first three weeks following the application of varnish with a gradual release of fluoride afterwards [80].

### 3.7. Fluoride Varnish for Caries Prevention

Several clinical trials were performed to evaluate the effect of fluoride varnish in caries prevention in both permanent teeth and primary teeth in children. These clinical studies have been subjected to several reviews, systematic reviews, and meta-analyses. Moreover, five Cochrane Library systematic reviews related to fluoride varnish application and caries prevention were conducted up to January 2021. It is evident from these meta-analyses and reviews that fluoride varnishes are considered caries inhibitory agents.

A Cochrane Library review was conducted in 2003 to address the effect of topical fluoride therapies (TFTs) in gel, toothpaste, mouth rinse, or varnish forms for dental caries prevention in children compared to not when no topical fluoride agent is used. The findings of the study revealed a significant caries increment reduction following topical fluoride therapy in both types of dentition. Two-thirds of children used topical fluoride in the form of toothpaste, followed by mouth rinses, gels, and varnish applications. A meta-analysis of 133 trials revealed that topical fluoride application on permanent dentition resulted in a reduction in caries of 26%. Only five studies mentioned the influence of topical fluoride therapy on caries increment on primary enamel surfaces. Two of these studies examined fluoride gel self-applications using a toothbrush. Three studies examined fluoride varnish applications. Those five studies were subjected to a meta-analysis, and the findings suggested that the use of topical fluoride applications in primary dentition is associated with a 33% reduction in decayed, missing, and filled tooth surfaces (dmfs) [81].

In a more recent Cochrane Library review conducted in 2013 on the effect of fluoride varnish in caries prevention in children compared to a placebo/or no treatment, 22 clinical trials were found. These included a total of 12,455 children who were randomized to either a study group that received fluoride varnish or to a control group that underwent no treatment or received a placebo. Thirteen trials were included in the meta-analysis. This analysis assessed the influence of FV on permanent teeth. The findings revealed that fluoride varnish application to permanent teeth is associated with a forty-three percent decrease in decayed, missing, and filled tooth surfaces. Ten trials were included in a meta-analysis that assessed the influence of fluoride varnish on the primary dentition. The findings suggested a 37% decrease in DMFS. The findings also revealed that the relative benefit of fluoride varnish application seems to occur regardless of the baseline severity of caries, the history of fluoride exposure (dentifrice, water supply, or other sources), the fluoride concentration used, the frequency of fluoride application, and whether the teeth had been subjected to prophylaxis prior to varnish application [19].

A recent systematic review done by Mishra et al. investigated the role of fluoride varnish in preventing early childhood caries. Studies published in the past 36 years were included, and the latest year of the search was 2015. Seventeen studies were included in the analysis. An analysis of the literature revealed that 1% and 5% concentrations of fluoride varnishes were used. The caries preventive fraction was found to range from 6.4% to 30% when 1% varnish was used and 5% to 63% when 5% fluoride varnish was applied [82].

### 3.8. Using Fluoride Varnish for Occlusal Caries Prevention

Pits and fissures are considered highly susceptible areas to caries. Fissures have large surface areas that allow more calcium fluoride to bind after fluoride varnish application [78]. Fluoride varnish is kept in close contact with the enamel for several hours or even days when compared with other forms of professional fluoride, such as gels [65]. Therefore, due to its prolonged adhesiveness and greater uptake of fluoride, varnishes are believed to be more effective for pit and fissure remineralization than other topical fluorides in both permanent and primary dentition [79].

In 1984, a study done by Holm et al. investigated the influence of Duraphat fluoride varnish (Colgate Oral Pharmaceuticals, New York, NY, USA) on the prevention of occlusal surfaces in newly erupted first permanent molars and compared this to a placebo (control group) in six-year-old children from a low-fluoride area. During a 2-year follow-up period, an examination of the first permanent molars was performed every three months using a probe and fissures were examined. Varnish was applied every six months. The caries incidence in the control group was 80%, while in the fluoride varnish group, the incidence was 35%. The caries preventive fraction was found to be 56% when varnish was used [83].

Another clinical trial was done by Tewari et al. to investigate the impact of topical application of APF, NaF, and Duraphat fluoride varnish on the prevention of pit and fissure caries in the molars, and this was compared with a control group (who received distilled water) of 6–12-year-old children from areas with low fluoridation. During a 2.5-year follow-up period, topical fluoride and Duraphat varnish were applied every six months. The Duraphat group showed a significantly higher caries reduction rate compared with the control group. The reduction rate was 70–75%. In contrast, the caries reduction rate was lower in the other two groups. It was 32–37% in the APF group and 20–33% in the NaF group. Their study suggested that Duraphat is the most potent topical fluoride agent in terms of preventing occlusal pit and fissure caries [84].

A recent Cochrane Library review compared fluoride varnish with sealants of different material types for different follow-up times to investigate their ability to prevent dental occlusal caries, and different results were found [85].

Three trials were included in the 2020 Cochrane Library review when glass ionomer-based sealants were compared with fluoride varnishes. One trial used a traditional chemically cured glass ionomer [86]. Two trials used a resin-modified glass ionomer [87,88]. It was not appropriate to conduct a meta-analysis because of the heterogeneity of the studies [85].

The study by Florio et al. assessed 21 children after a one-year follow-up period and revealed no differences between those who were treated with fluoride varnish versus resin-modified glass ionomer sealant in terms of occlusal caries development [87].

However, in the study conducted by Tagliaferro et al. in 2011, which compared the effect of resin-modified glass ionomer sealant with that of fluoride varnish in terms of occlusal caries prevention over a 2-year follow-up period for children with a high caries risk, sealant application accompanied by oral health education was superior to the application of fluoride varnish for occlusal caries prevention [88].

In the third glass ionomer study, the influence of fluor protectors (Ivoclar Vivadent, Schaan, Liechtenstein) in occlusal caries prevention was compared with traditional chemically cured glass-ionomer cement used as a pit and fissure sealant after 3 years. The results showed that the incidence of caries was lower in the sealant group and the FV group than in the control group that underwent no treatment, and the difference was significant (*p* < 0.01). However, no significant difference was found between the two experimental groups [86].

Five trials included in the 2020 Cochrane Library review evaluated the effect of fluoride varnish compared with resin-based sealants for occlusal caries reduction [46,89,90,91,92].

Four out of the five studies assessed the first permanent molars after a 2–3-year follow-up period and were subjected to a meta-analysis [46,89,90,91]. The results of the meta-analysis were inconclusive as to whether fluoride varnishes are superior to resin fissure sealants in preventing occlusal caries or vice versa [85].

One of the studies included in the 2020 Cochrane Library review was a Spanish study conducted by Bravo in 2005. This study involved follow-up testing after 4 years and 9 years (it was started in 1990), in which resin sealants were placed on sound fissures and were compared with fluoride varnish. Reapplication of sealants took place when they were totally or partially lost during the 4-year active preventive program. The results revealed that resin sealants were preferable to FV at both the 4-year and 9-year follow-up points. The caries incidence on occlusal surfaces was 77% in the control group, 26.6% in those with resin-sealed teeth, and 55.8% in those with fluoride-varnished teeth after 9 years. However, due to the high drop-out rate (67%) at the 9-year test point, the results lack reliability [91].

One German split-mouth trial that compared resin sealant with fluoride varnish versus fluoride varnish alone at a two-year follow-up point showed that the simultaneous application of resin sealant and fluoride varnish had a significantly superior effect on occlusal caries reduction compared with fluoride varnish alone. Children were examined biannually for 24 months. At the time of examination, FV was painted on all teeth, including the teeth that received sealant. Additionally, sealants were reapplied if lost. Research authors declared that the study was done in a population with low caries risk [85].

A more recent clinical trial compared the effectiveness of resin-based fissure sealant with Duraphat FV (Colgate Oral Pharmaceuticals, New York, NY, USA) for the prevention of dental caries on the occlusal surfaces of first permanent molars in 6 to 7-year-old school children. Both interventions were applied to sound enamel and were re-evaluated every 6 months. The fluoride varnish was reapplied every 6 months for 3 years. Fissure sealants were reapplied if they were lost completely or if fissures were incompletely covered as a result of additional tooth eruption or partial loss of the sealant. The study reported no significant difference between the two intervention groups in terms of the number of teeth that developed dental caries. These findings suggested that in community oral health programs targeting high caries risk children, after three years, the application of fluoride varnishes twice per year as a caries preventive strategy will prevent occlusal caries to the same extent as the application of fissure sealants [90].

A recent study done by Cabral et al. investigated the caries preventive effect and retention rate of a light-curable RMGI varnish (Vanish™ XT Extended Contact Varnish, 3M ESPE, St. Paul, MN, USA) and a high-viscosity GIC used as a sealant on recently erupted permanent first molars after 24 months. The effectiveness of the light-curable RMGI varnish in caries prevention was found to be equal to that of the high-viscosity GIC, but the latter was shown to have a longer retention rate than the RMGI varnish [93].

## 4. Conclusions

Following a wide range of clinical trials with a vast variety of methodologies and results, fluoride varnishes have been shown to be effective for preventing occlusal caries in children with a moderate to high risk of caries. Both fluoride varnish and resin-based fissure sealants were found to be effective for preventing occlusal caries to the first permanent molars, but it was not possible to identify which type of treatment is superior. The application of fluoride varnish should be based on risk assessment. Fluoride varnish should be applied two to four times per year.

## Figures and Tables

**Table 1:** Summary of the two main types of fluoride varnish.

	Sodium Fluoride	Fluoride Silane
Commercial name	Duraphat, Colgate Oral Pharmaceuticals, New York, NY, USA	Fluor Protector, Ivoclar Vivadent, Schaan, Liechtenstein
Discovered	in 1960	in 1975
Main component	5% sodium fluoride (NAF) by weight	0.9% difluorosilane by weight
Fluoride concentration ppm	22,600 ppm F	1000 ppm F
Fluoride concentration %	2.26% fluoride	0.1% fluoride
Type	Resin base varnish	polyurethane-based varnish
Available as	10 mL tube suitable for multiple uses	either a 0.4 mL single use or a 1.0 mL ampule for multiple doses
Mechanism of action	-Bacterial inhibition -Fluroapitate formation -Inhibition of demineralization -Enhancing remineralization	-Bacterial inhibition -Fluroapitate formation -Inhibition of demineralization -Enhancing remineralization
